# Real-time PCR complements immunohistochemistry in the determination of HER-2/neu status in breast cancer

**DOI:** 10.1186/1472-6890-6-2

**Published:** 2006-01-18

**Authors:** Andreea Nistor, Peter H Watson, Norman Pettigrew, Karim Tabiti, Angelika Dawson, Yvonne Myal

**Affiliations:** 1 Department of Pathology, University of Manitoba, 770 Bannatyne Ave., Winnipeg, Manitoba R3E 0W3, Canada; 2 Department of Pathology, Immunopathology Laboratory, Health Sciences Centre, 820 Sherbrook St., Winnipeg, Manitoba R3A 1A9, Canada; 3 Head Alliance Management Oncology, Clinical Genomics, Roche diagnostics GmBH, Nonnenwaldstr. 2, D-82372 Penzberg, Germany; 4 Department of Biochemistry & Medical Genetics, University of Manitoba, 770 Bannatyne Ave., Winnipeg, Manitoba R3E 0W3 and the Cytogenetics Laboratory, Health Sciences Centre 820 Sherbrook St., Winnipeg, Manitoba R3A 1A9, Canada; 5 Molecular Diagnostic Pathology Laboratory, Department of Pathology, Health Sciences Centre, 820 Sherbrook St., Winnipeg, Manitoba R3A 1A9, Canada

## Abstract

**Background:**

The clinical benefit of determining the status of HER-2/*neu *amplification in breast cancer patients is well accepted. Although immunohistochemistry (IHC) is the most frequently used method to assess the over-expression of HER-2 protein, fluorescent *in-situ *hybridization (FISH) is recognized as the "gold standard" for the determining of HER-2/*neu *status. The greatest discordance between the two methods occurs among breast tumors that receive an indeterminate IHC score of 2+. More recently, a real-time polymerase chain reaction (PCR) assay using the LightCycler^® ^has been developed for quantifying HER-2/*neu *gene amplification. In this study, we evaluated the sensitivity and specificity of a commercially available LightCycler assay as it compares to FISH. To determine whether this assay provides an accurate alternative for the determination of HER-2/*neu *status, we focused primarily on tumors that were deemed indeterminate or borderline status by IHC.

**Methods:**

Thirty-nine breast tumors receiving an IHC score of 2+ were evaluated by both FISH and LightCycler^® ^technologies in order to determine whether quantitative real-time PCR provides an accurate alternative for the determination of HER-2/*neu *status.

**Results:**

We found a high concordance (92%) between FISH and real-time PCR results. We also observed that 10% of these tumors were positive for gene amplification by both FISH and real-time PCR.

**Conclusion:**

The data show that the results obtained for the gene amplification of HER-2/*neu *by real-time PCR on the LightCycler^® ^instrument is comparable to results obtained by FISH. These results therefore suggest that real-time PCR analysis, using the LightCycler^®^, is a viable alternative to FISH for reassessing breast tumors which receive an IHC score of 2+, and that a combined IHC and real-time PCR approach for the determination of HER-2 status in breast cancer patients may be an effective and efficient strategy.

## Background

Gene amplification and over-expression of the HER-2/*neu *gene, also known as c-*erb*B-2 or *ERBB2*, is frequently observed (approximately 25–30%) in human breast cancer [1]. The HER-2/*neu *gene, which is a member of the epidermal growth factor receptor (HER) family, is located on chromosome 17q11.2-12 [2] and encodes a 185 kDa transmembrane tyrosine kinase receptor protein.

Considered to play a role in the biologic behavior or pathogenesis of human breast cancer, the amplification of the HER-2/*neu *gene is now regarded as an established predictive [3] and prognostic [4] marker for breast cancer, particularly for the management of advanced breast cancer. Both node-positive and node-negative breast cancer patients whose tumors exhibit HER-2/*neu *amplification, have a poor prognosis, an increased risk of recurrence and a high risk of disease-related death showing overall shorter survival rates [5-10].

However, the major interest in HER-2/*neu *amplification lies in its utility as a predictive marker of responsiveness to therapy [11] primarily, the response of breast cancer patients to chemotherapy, hormonal therapy (antiestrogens) and therapeutic anti-HER-2 antibodies. Tumors with amplification of this oncogene are less responsive to CMF (cyclophosphamide, methotrexate, and 5-fluorouracil) adjuvant chemotherapy regimens than those with a normal levels of the gene product [12]. Conversely, HER-2/*neu *amplification is a useful marker to identify the patients who are most likely to benefit from high doses of doxorubicin (Adriamicin) therapy [13-15]. Patients with HER-2/*neu *gene amplification/over-expression are less responsive to tamoxifen therapy [16-18]. However, the HER-2/*neu *status is mainly used for identifying patients with advanced breast cancer who may benefit from the therapy with anti-HER-2 antibody trastuzumab/Herceptin^® ^(Genetech, San Francisco, CA) a humanized murine monoclonal antibody which has been shown to be effective in prolonging survival in patients with receptor positive metastatic breast carcinoma [19].

While the clinical benefit of determining the status of HER-2/*neu *amplification/over-expression is clearly accepted, several methods for assessment and quantification of HER-2/*neu *gene alteration have been used in the search of an accurate, quantitative, widely applicable in clinical setting and cost-effective assay.

Immunohistochemistry (IHC) is most frequently used to assess the over-expression of HER-2 protein. It is an indirect method of measuring gene amplification and is based on the ability of antibodies to identify HER-2 proteins expressed by fixed cells in frozen or paraffin embedded tissue sections. In general, IHC is carried out on paraffin embedded tissues using standard laboratory equipment and therefore is the method of choice for most clinical laboratories. However, IHC analysis relies on subjective interpretation of staining intensity and extent of tumor cells within a section in order to assign an expression score of 0/1+ (regarded as IHC negative) or 2+/3+ (regarded as IHC positive).

Determination of HER-2/*neu *amplification by Fluorescent *in-situ* hybridization (FISH) strategies is an alternate method of choice. Whereas IHC detects the over-expression of the HER-2 protein, FISH is a direct method to detect HER-2/*neu *gene amplification. However, FISH is more time consuming to perform, relatively expensive and requires specialized equipment. Nevertheless, the accuracy of this technique means that FISH assessment is accepted as the "gold standard" for the determination of HER-2/*neu *status in paraffin embedded breast tumors.

Polymerase chain reaction (PCR)-based assays are able to determine both changes in HER-2/*neu *gene number and expression [20]. Real-time PCR analysis of DNA offers a precise quantitative analysis of gene amplification. The advantage of this technique is that it is simple, convenient, non-radioactive and rapid so that it is highly suitable for a routine clinical laboratory. No expertise beyond accurate and aseptic pipetting technique is required. However, results from real-time PCR quantitative assays can be affected by contamination of the tumor sample with nucleic acids from non-tumor surrounding tissues. This problem can be reduced by using laser-assisted microdissection, or if not available, by carefully outlining the tumor area on the slide and using only the tumor tissue for analysis.

The evaluation of HER-2/neu status at the mRNA level by reverse-transcription PCR (RT-PCR) has also been proposed [20,21] but the use of formalin-fixed paraffin-embedded tissue for this purpose is problematic as the RNA is often extensively degraded. Therefore, the use of RT-PCR is limited to availability of frozen tissue.

Chromogenic *in situ *hybridization (CISH) is a more recently introduced method for detecting HER-2/*neu *amplification [22]. CISH makes use of the *in situ *hybridization technology but also takes advantage of the chromogenic signal detection of IHC. With CISH, a DNA probe is detected using a simple IHC-like peroxidase reaction and positive signals can be detected with the ordinary light microscope. One advantage of CISH is that the assay is one quarter the cost of FISH and can be used when fluorescent microscopy is not available. However CISH, like IHC, relies on subjective interpretation and the utility of some low/high amplified CISH results are sometime in question.

To date, the FDA has approved two methods [23] for the selection of cancer patients for receiving Herceptin^® ^(Trastuzumab) therapy: IHC and FISH. In practice, both techniques are used in conjunction. IHC assay is used as an initial screening assay to identify clearly negative or positive cases. The FISH assay is then used subsequently in those cases where IHC status is indeterminate (2+ IHC positive cases). This sequential strategy avoids the high cost and relatively longer time delay of using the FISH assay in all cases. More recently, a LightCycler HER-2/*neu *DNA Quantification kit (Roche Molecular Biochemicals, Mannheim, Germany) is currently commercially available and a real-time PCR LightCycler assay has been developed for HER-2/*neu *evaluation. Advances in PCR technology using the real-time fluorescent monitoring capabilities of the LightCycler have the potential to enable relatively inexpensive, rapid and accurate quantification of gene amplification [24]. The evaluation of the quantitative real time PCR assay for HER2/*neu *is the focus of this study.

Our objective was to evaluate the specificity and sensitivity of the LightCycler real-time PCR assay as it compares to FISH, focusing primarily on tumors that are indeterminate or borderline HER-2 status by IHC.

## Methods

### Tissue samples

Between January 2001 and December 2004, 1673 breast tumors were routinely examined by IHC analysis for HER-2 protein overexpression at the Immunopathology Laboratory at the Health Science Center, Winnipeg, Manitoba. The tumor samples were fixed, processed and paraffin embedded according to standard protocols [25]. Borderline samples (scored +2 by IHC) consisted of approximately 10% of the total number of breast tumor samples tested.

Representative Paraffin blocks of 39 randomly selected primary breast tumor samples ranked +2 by IHC were obtained from the Immunopathology Laboratory. FISH and Real-Time PCR analysis were conducted by the Molecular Diagnostic Pathology Laboratory. For real-time PCR analysis, tumor areas with a minimum 100 tumor cells/cm^2 ^and minimal 'contaminating' normal breast tissue were outlined on the cover glass slip with a permanent marker. The outlined areas were scraped into eppendorf tubes for DNA extraction.

This study was approved by the Pathology Access Committee for Tissues (PACT) and the Research Ethics Board at the University of Manitoba.

### Immunohistochemistry (IHC) analysis

IHC analysis of the HER-2 protein expression was carried out in histologic sections of breast cancer specimens using the HercepTest (DAKO, CA, USA). The HercepTest was approved by the FDA (September, 1988) for selection of women with breast cancer in order to receive trastuzumab humanized monoclonal antibody therapy. Six μm thick tissue sections of the paraffin-embedded blocks were cut, mounted on silane-coated slides, deparaffinized, heat-treated for antigen retrieval [26] and immunostained. The heat treatment involved heating in pressure cooker in 10 mmol/L citrate buffer for 40 minutes and tissue sections were then cooled. The immunostaining process was carried out using the DAKO Autostainer Universal Staining System according to the instructions of the manufacturer (DAKO, Corp). Briefly, tissue sections were treated with peroxidase-blocking reagent for 5 minutes, rinsed and treated with rabbit anti-human HER-2 primary antibody for 30 minutes. The sections were then rinsed and treated for 30 minutes with secondary goat anti rabbit antibody and horseradish peroxidase and, following rinsing, incubated in diaminobenzidine for 10 minutes. The sections were removed from the Autostainer and counterstained with hematoxylin and mounted in Permount. Membrane staining was interpreted as HER-2 protein overexpression using a bright-field Olympus microscope according to an established scoring system [20] as 0, 1+, 2+ and 3+ as follows: 0 indicating absence of staining, 1+ indicating the lowest level of detectable staining and/or non-homogenous weak staining, 2+ indicating moderate homogenous membrane staining and 3+ indicating intense homogenous membrane staining. Immunostaining was considered positive when more than 10% of all cells had 2+ or 3+ staining intensity.

### FISH analysis

FISH analysis was carried out using the Vysis LSI HER-2/*neu *SpectrumOrange and CEP 17 SpectrumGreen Dual Color DNA probe kit (Vysis PathVysion^®^, Abbot Laboratories, IL) according to the manufacturer's instructions. The locus specific identifier (LSI) HER-2/neu DNA probe is a 190-Kb SpectrumOrange fluorescent-labeled probe that specifically hybridizes to the HER-2/neu gene locus, 17q11.2-q12. The chromosome enumeration probe (CEP) 17 is a 5.4-Kb SpectrumGreen fluorescent labeled DNA probe specific for the alpha satellite DNA sequence at the centromeric region of chromosome 17, 17p11.1-q11.1.

HER-2/*neu *gene copy level was determined by FISH analysis of paraffin-embedded tissue sections as a ratio between the HER-2/neu gene copies and the chromosome 17 centromere copies (Vysis, Inc.). This approach excludes polysomy of chromosome 17 as a source of increased HER-2/*neu *gene copy number.

The 6 μm thick tissue sections on slides were deparaffinized in xylene, followed by dehydration with absolute ethanol, air-dried and pre-treated using the Vysis Paraffin Pretreatment Kit I (Vysis, Inc.). Following pre-treatment, the hybridization process was performed at 37°C for 14–18 hours (overnight) and the nuclei were then counterstained with 4'-6'-diamidino-2'-phenylindole (DAPI). Enumeration of HER-2/*neu *and CEP 17 probe signals was performed under fluorescence microscopy with a 100-watt mercury lamp at 1000X magnification using a Zeiss Axiophot fluorescent microscope with triple bands filters.

Scoring was restricted to cancer cells by demarcation of the tumor area on the H&E stained slides by two breast cancer pathologists (PW, WO). In each specimen, 60 non-overlapping nuclei were counted. The green signals for centromere 17 and the orange signals for HER-2/*neu *gene were recorded and the ratio calculated for each slide. According to the manufacturer's guidelines, nuclei without signals or only one-color signals or nuclei with insufficient DAPI counter-stain to determine the nuclear border were not scored. A ratio of HER-2/*neu *to chromosome 17 copy number ≥ 2.2 was considered positive and a ratio < 1.8 was considered negative for HER-2/*neu *amplification. The scoring of slides, which presented a ratio between 1.8 and 2.2, was re-evaluated.

### Real-Time Quantitative PCR Analysis of HER-2/neu Gene Amplification

The DNA extraction was performed using the High Pure PCR Template Preparation Kit (Roche).

Real-Time PCR Analysis was performed using the LightCycler (Roche Molecular Biochemicals, Mannheim, Germany) and the LightCycler HER-2/*neu *Quantification Kit (Roche Molecular Biochemicals, Mannheim, Germany). The gastrin gene was used as the reference housekeeping gene. A 112-bp fragment of the Her-2/neu gene and a 133-bp fragment of the gastrin gene were amplified during the PCR reaction. The simultaneously quantification of the HER-2/*neu *gene and the reference gene that serves both as control for DNA integrity and as a reference for relative quantification was achieved by using different labeled hybridization probes (Red-705 for the HER-2/*neu *and Red-640 for the gastrin specific oligonucleotide) which allow dual color detection in the same capillary. Five μl (approximately 1 μg) of each sample of the template DNA was mixed with the ready-to-use primers and hybridization probes, enzyme solution (Taq DNA polymerase) and dNTP reaction mix included in the kit in a final volume of 20 μl. The pre-incubation and denaturation of the template DNA were performed at 95°C for 12 minutes. This was followed by amplification of the target DNA through 45 cycles of denaturation at 95°C for 10 seconds, annealing at 58°C for 10 seconds and elongation at 72°C for another 10 seconds. The specimens were then cooled to 40°C. Samples were analysed in duplicates. The LightCycler HER-2/*neu *Quantification Kit contained a calibrator DNA provided to generate a calibration curve. The crossing point data for the HER-2/*neu *and the reference gene were determined and exported into Relative Quantification Software that allowed the calculation of the HER-2/gastrin normalized ratio. A ratio target gene/reference gene = 2.0 was considered positive for HER-2/*neu *gene amplification.

### Statistical analysis

Statistical analysis was performed using GraphPad PRISM^®^, Version 3.02. The concordance between Real Time LyghtCycler PCR and FISH assays were analysed by a Fisher's exact probability test (Table 2), the cut-off point to assign negative and positive status for each method being established as previously described in Material and Methods. The correlation between the two tests was established by calculating the Spearman correlation coefficient.

## Results

**Figure 1:**
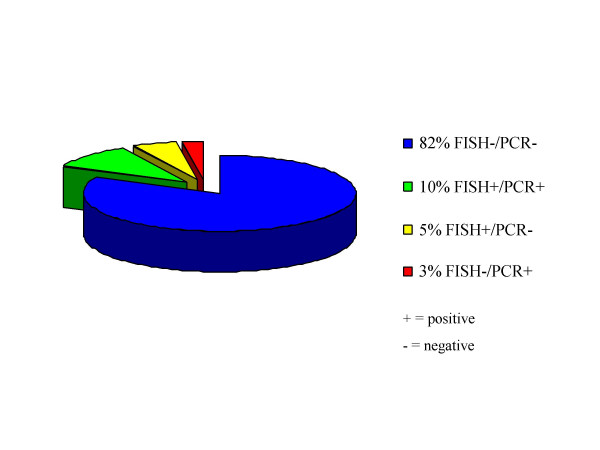
Concordance between FISH and real-time PCR in IHC 2+ cases (92%).

As FISH is currently the "gold standard" method for evaluation of HER-2/*neu *amplification we wanted to examine the performance of the LightCycler Real Time PCR assay as measured against FISH assay in IHC borderline cases. Thirty-nine breast tumor samples that scored 2+ for HER-2 protein expression by IHC analysis were selected for this study. Our results (summarized in Table 1) show that of the 39 tumors, 32 (82%) were scored negative for HER-2/*neu *gene amplification and 4 (10%) were scored positive by both PCR and FISH methods, the overall correlation between FISH and real-time PCR results being 92% (36 of 39 tumors). Of the remaining 3 tumors, 1 was positive by real-time PCR and negative by FISH and 2 were positive by FISH and negative by PCR (Fig. 1).

The results of our study are summarized in the contingency table (illustrated in Table 2) generated by categorizing the results according to the cut-off points described in Material and Methods for each test. Statistical analysis confirmed the 92% concordance between the HER-2/neu statuses assessed by the 2 methods (p < 0.0009, two-sided Fisher's test). The Spearman rank correlation coefficient was r = 0.45 at 95% confidence interval (p = 0.0038, two-tailed Spearman correlation).

## Discussion

Thirty percent of breast cancer patients exhibit overexpression of the HER-2 gene and the HER-2/*neu *status correlates with the response to both specific receptor targeted therapy as well as other treatments. It has therefore been recommended that routine screening for HER-2/*neu *amplification should be performed as part of the evaluation of each new diagnosed case of breast carcinoma [27]. Whereas a lack of assessment of the HER-2/*neu *status deprives breast cancer patients from beneficial treatment, a false positive diagnosis of gene amplification could lead to an unnecessary expensive therapy with multiple possible side effects, including cardiac dysfunction [28].

In order to establish the best strategy to assess the HER-2/*neu *status, numerous studies have compared the results of the two FDA approved assay methods. The discordance between the accuracy of HER-2 protein as determined by IHC and gene amplification as determined by FISH has sparked much debate in the last few years. Some authors have reported a concordance rate as high as 91% [29] or 88% [30], but claim a weak concordance between the two methods when the HercepTest score is 2+, such as a 35% concordance [30]. Similarly, 75% of the discordant cases (positive by IHC and negative by FISH) had a HercepTest score of 2+ [31]. On the other hand, in a large recently published study [32], only 0.7% of the IHC negative tumors were found to be positive by FISH and 5.9% IHC 3+ tumors were FISH negative so that there was a very good correlation between the HER-2/neu status assessed by FISH and IHC in 0, 1+ as well as 3+ tumors. Therefore it has been proposed that the 2+ score, as defined in the guidelines for the FDA-approved HercepTest, should not be used as a criterion for trastuzumab therapy unless confirmed by FISH [33].

Our study was therefore focused predominantly on this borderline 2+ IHC category. Using a real-time PCR quantitative assay, we found a 92% concordance between FISH and real-time PCR results in breast tumors previously assessed with moderate increased HER-2 protein expression (2+) by IHC. We also found that 10% of these tumors were positive for gene amplification by both FISH and real-time PCR, and the remaining 90%, determined to be negative. The high concordance (92%) between the FISH and real-time PCR results as well as the discordance between both of these 2 methods with the IHC assay, suggests that real-time PCR is more accurate in determining the patients who are true candidates for trastuzumab therapy. A similarly high concordance between FISH and real-time PCR was previously reported [34,35]. In addition, the same authors [34] reported that the observed 92% concordance rate later increased to 98% following the incorporation of laser-assisted microdissection into the real time PCR protocol, and they were then able to reclassify the FISH positive, PCR negative specimens to FISH positive, PCR positive.

We also found that two cases negative by FISH, exhibited polysomy of chromosome 17. Interestingly, in these two tumor samples, IHC detected the increased HER-2 protein expression but could not discriminate between true overexpression and polysomy of chromosome 17. We recognize that polysomy of chromosome 17 can occur in a detectable frequency. Therefore, for quantitative real time PCR assays, consideration should be given to the introduction of a second control gene that is located at a distance away from the amplifiable region and able to detect numerical abnormalities of chromosome 17. The determination of the copy number of chromosome 17 might be helpful in differentiating breast cancer patients with polysomy of chromosome 17 and those that overexpress the of HER-2 protein and may help identify subgroups of patients that probably have genetic and clinical differences [21,36].

It is also important to note that the prevalence of cancer cells in scraped tissue used for DNA extraction or the inclusion of microdissection strategies, will have an impact on the real-time PCR results. Thus, samples previously deemed PCR negative could be truly PCR positive, when microdissection of the pure tumor tissue was employed.

The results of our present study demonstrate that there can be discordance between the most commonly used assay method (IHC) and other methods of assessing the HER-2 status. Specifically in IHC borderline positive cases, our data suggest that real-time PCR possesses a high potential to enhance accuracy in clinical settings for diagnosis of true trastuzumab therapy candidates. These data need to be confirmed by a larger study as well as a clinical study designed to compare how the HER-2/*neu *status assessed by FISH and real-time PCR correlates with the response to Herceptin therapy.

## Conclusion

The results obtained by real-time PCR for the amplification of HER-2/*neu *gene in breast tumors were comparable with results obtained by FISH and suggests that real-time PCR using the LightCycler is a viable alternative to FISH for evaluating tumors deemed indeterminate by IHC. A combined IHC and real-time PCR approach for determining HER-2/*neu *amplification in breast cancer patients may be an effective and efficient strategy.

## Competing interests

The author(s) declare that they have no competing interests.

## Authors' contributions

Andreea Nistor analyzed the data, carried out statistical analysis and wrote the manuscript.

Yvonne Myal generated the idea for the study and supervised the study.

Peter Watson provided intellectual input and critically revised the manuscript.

Norman Pettigrew supplied tumor specimens and was responsible for IHC analysis.

Angelika Dawson provided advice and interpretation of FISH analysis.

Karim Tabiti assisted with the interpretation of the LightCycler data.

## Pre-publication history

The pre-publication history for this paper can be accessed here:



## Figures and Tables

**Table 1:** Comparison of HER-2/*neu *status assessed by FISH and Real-Time PCR in 39 breast cancer samples deemed 2+ by IHC

**Sample number**	**FISH**				**Real-Time PCR**	
	
	**CEP 17**	**HER-2**	**Ratio**	**Status**	**Ratio**	**Status**
1	1.83	1.83	1.00	-	1.33	-
2	1.45	1.60	1.10	-	0.48	-
3	1.18	1.23	1.04	-	0.65	-
4	1.35	1.27	0.94	-	1.3	-
5	1.48	1.75	1.18	-	1.5	-
6	1.37	1.37	1.00	-	1.74	-
7	2.40	3.21	1.34	-	0.66	-
8	1.88	1.73	0.92	-	1.35	-
9	6.22	5.85	0.94	-	1.46	-
10	1.77	1.75	0.99	-	1.62	-
11	1.28	1.28	1.00	-	1.49	-
12	1.72	1.68	0.98	-	1.52	-
13	1.53	1.50	0.98	-	1.42	-
14	1.80	1.78	0.99	-	1.43	-
15	3.30	3.22	0.98	-	1.18	-
16	2.47	2.45	0.99	-	1.59	-
17	1.73	1.82	1.05	-	0.41	-
18	1.70	1.73	1.02	-	1.53	-
19	1.83	1.93	1.05	-	0.35	-
20	3.03	3.55	1.17	-	1.58	-
21	1.97	1.55	0.79	-	1.65	-
22	1.68	1.72	1.02	-	1.60	-
23	1.40	1.20	0.86	-	1.39	-
24	1.27	1.80	1.42	-	1.77	-
25	1.20	1.18	0.98	-	1.68	-
26	1.07	1.07	1.00	-	0.44	-
27	1.10	1.08	0.98	-	0.76	-
28	1.98	2.40	1.21	-	1.49	-
29	1.85	2.58	1.39	-	1.96	-
30	2.70	3.22	1.19	-	1.76	-
31	1.95	1.97	1.01	-	1.84	-
32	4.17	6.85	1.64	-	1.67	-

**33**	**1.92**	**2.00**	**1.04**	**-**	**2.07**	**+**
**34**	**1.77**	**7.13**	**4.03**	**+**	**1.83**	**-**
**35**	**1.93**	**4.92**	**2.55**	**+**	**1.51**	**-**

36	1.92	6.77	3.53	+	3.91	+
37	1.77	16.70	9.44	+	11.10	+
38	2.00	5.87	2.94	+	2.09	+
39	2.00	8.15	4.08	+	2.39	+

CEP 17 = mean/cell of the chromosome 17 centromere probe signals HER-2 = mean/cell of the HER-2/neu probe signals

FISH Ratio = the ratio between HER-2 and CEP 17

Real-Time PCR Ratio = the normalized ratio between HER-2 and the reference gene (gastrin)

"+" = positive, "-"=negative. Cases (8%) that were discordant between PCR and FISH assays are highlighted.

**Table 2:** Contingency table showing the breast tumors included in our study categorized according to the cutoff points established for each test (as described in Material and Methods).

**FISH\PCR**	-	+
-	32	2
+	1	4
